# DNA double-strand breaks with 5′ adducts are efficiently channeled to the DNA2-mediated resection pathway

**DOI:** 10.1093/nar/gkv969

**Published:** 2015-09-29

**Authors:** Margaret Tammaro, Shuren Liao, Neil Beeharry, Hong Yan

**Affiliations:** Fox Chase Cancer Center, 333 Cottman Avenue, Philadelphia, PA 19111, USA

## Abstract

DNA double-strand breaks (DSBs) with 5′ adducts are frequently formed from many nucleic acid processing enzymes, in particular DNA topoisomerase 2 (TOP2). The key intermediate of TOP2 catalysis is the covalent complex (TOP2cc), consisting of two TOP2 subunits covalently linked to the 5′ ends of the nicked DNA. In cells, TOP2ccs can be trapped by cancer drugs such as etoposide and then converted into DNA double-strand breaks (DSBs) that carry adducts at the 5′ end. The repair of such DSBs is critical to the survival of cells, but the underlying mechanism is still not well understood. We found that etoposide-induced DSBs are efficiently resected into 3′ single-stranded DNA in cells and the major nuclease for resection is the DNA2 protein. DNA substrates carrying model 5′ adducts were efficiently resected in *Xenopus* egg extracts and immunodepletion of *Xenopus* DNA2 also strongly inhibited resection. These results suggest that DNA2-mediated resection is a major mechanism for the repair of DSBs with 5′ adducts.

## INTRODUCTION

DNA double-strand breaks (DSBs) are among the most deleterious damages to the genome. They may arise exogenously by environmental agents, such as ionizing radiation or chemotherapeutic drugs, and endogenously from replication fork breakage, V(D)J recombination, or SPO11-mediated cleavage during meiosis (1–4). Many cancer-prone genetic disorders, such as Werner syndrome and Bloom syndrome, are defective in DSB repair. Clinically, some of the mainstay cancer treatments, such as the topoisomerase inhibitors etoposide and camptothecin, act by inducing DSBs in cells.

There are two major mechanisms for the repair of DSBs: non-homologous end joining (NHEJ) and homology-dependent repair (HDR) (3,5–7). NHEJ is intrinsically error-prone, but active throughout the cell cycle. HDR is generally error-free, but is active only during S and G2 phases. The key event in the bifurcation of NHEJ and HDR is the initial processing of DNA ends. While NHEJ involves limited processing, HDR requires extensive processing to form long 3′ ss-tails. Recent studies in several model systems have revealed that there are two major pathways for the degradation of the 5′ strand of DNA (8). One pathway is catalyzed by the combined actions of a RecQ-type DNA helicase (such as the Werner syndrome protein, Sgs1, and the Bloom syndrome protein), the DNA2 5′->3′ ss-DNA exonuclease and the ss-DNA binding protein replication protein A (RPA) (9–14). The other pathway is catalyzed by a 5′->3′ ds-DNA exonuclease EXO1 (12,13,15). Both pathways are initiated by the RMX/MRN (MRE11-RAD50-XRS2/NBS1) complex and SAE2/CtIP (12,13,16–19).

The current understanding of DSB repair is mostly based on simple DNA ends generated by sequence-specific endonucleases, but DSBs generated in cells are often complicated in structure. Of particular importance, both biologically and clinically, is the type of DSBs carrying adducts at the 5′ end. During meiosis, DSBs are deliberately introduced by the SPO11 nuclease, which then stays linked to the 5′ end of the break, to induce recombination between homologous chromosomes (20). Some linear ds-DNA viruses, such as adenovirus, have a terminal protein covalently attached to the 5′ end to prime DNA synthesis (21). In normal cells, the major source of DSBs with 5′ adducts is trapped DNA topoisomerase 2 (TOP2) (22,23). TOP2 is a dimeric enzyme that changes the topology of DNA and thus plays essential roles in diverse DNA transactions such as replication, transcription, chromosome condensation and chromosome segregation (22–24). It acts by nicking the two strands of DNA to form a gate, then directing another DNA molecule to pass through the gate and finally resealing the nicks to close the gate. A key intermediate in the catalytic cycle is the cleavable complex (TOP2cc), a covalent complex linked by a phosphodiester bond between the tyrosine at the catalytic center of each TOP2 subunit and the 5′ end of each nicked DNA strand. TOP2ccs are normally transient in existence but can be trapped by nearby DNA lesions, natural metabolites, or, most importantly from the clinical point of view, cancer drugs such as etoposide that bind to TOP2 (23). The two subunits reseal the nicks independently of each other, so trapped TOP2ccs can be either single-stranded or double-stranded (25). In cells, trapped TOP2ccs, even the double-stranded ones, are not directly sensed as DSBs due to the strong interaction between the two subunits that can hold the two sides of the DSB together (26). They are converted into true DSBs after collision with the transcription machinery or the replication fork complex (27). Transcription stimulates the degradation of trapped TOP2ccs, a ds-TOP2cc is thus converted into a true DSB with a degraded TOP2 at the 5′ end. Replication run-off, on the other hand, can convert a ss-TOP2cc into a DSB with a TOP2 at the 5′ end (28–30). While there is no direct evidence due to technical difficulties, the existence of DSBs with degraded or intact 5′ TOP2 *in vivo* is nevertheless supported by many observations. In particular, an enzyme, tyrosyl-DNA phosphodiesterase 2 (TDP2), has been identified to specifically cleave off the degraded (to small peptides or the catalytic tyrosine) or denatured (intact size) TOP2 located at the 5′ end but not internally (31,32). Consistent with the enzymatic activity, mutant cells lacking TDP2 are highly sensitive to etoposide (31,33). This not only supports a critical role for TDP2 in repairing trapped TOP2ccs but also confirms the prediction that degraded or denatured TOP2 is located at the 5′ end of DSBs. There is additional evidence for the existence of intact or largely intact TOP2 at the 5′ end of DSBs. Yeast SAE2 is known to activate a cryptic endonuclease activity in MRE11 to nick the 5′ strand at a position close to the end, but the activation is absolutely dependent on the presence of a bulky terminal adduct such as streptavidin (34). Using anti-TOP2 antibodies as the probe, it has been found that intact or largely intact TOP2 trapped on DNA in etoposide-treated cells can be efficiently released by a complex containing MRN (34). This is consistent with the prediction that intact or largely intact TOP2 is located at the 5′ end rather than internally of DNA.

The repair of DSBs with bulky adducts such as degraded or intact TOP2 at the 5′ end poses new problems to the DSB repair machineries (22). NHEJ and TDP2 are known to play major roles in repairing etoposide-induced DSBs. Genetic analysis shows that TDP2 and Ku70 are epistatic to each other such that TDP2 ku70 double mutant cells and ku70 single mutant cells have the same sensitivity to etoposide (35). This suggests that after the removal of the degraded TOP2 by TDP2, the resulting clean ends are channeled to the NHEJ machinery. In addition to NHEJ, there is also evidence for homology-dependent pathways in repairing etoposide-induced DSBs in human cells (36). However, unlike the TDP2-mediated NHEJ, the homology-dependent repair mechanism for etopside-induced DSBs is still poorly understood. Etoposide might induce not only DSBs with 5′ degraded or intact TOP2 but also DSBs with clean ends (if a replication fork approaches from the 5′ side). As such it is unclear if in etoposide-treated cells HDR is used to repair DSBs with 5′ adducts or DSBs with clean ends. Similarly, while MRN/MRX and CtIP/SAE2 can remove 5′ adducts from DSBs, there is no evidence that these processed DSBs are then channeled to resection instead of NHEJ.

In this study we used a combination of cell biological methods and biochemical reconstitution to investigate the mechanism of homology-dependent repair of DSBs with 5′ adducts. We found that etoposide-induced DSBs are efficiently resected into 3′ ss-DNA in cells and that the DNA2 protein is a major nuclease for resection. Consistent with this function, cells depleted of DNA2 are hypersensitive to etoposide. Biochemically, we found that model substrates carrying various types of 5′ adducts are efficiently resected in *Xenopus* egg extracts and these resection reactions are strongly inhibited by the depletion of DNA2. These results show that DNA2-mediated resection is a major pathway for the repair of DSBs with 5′ adducts.

## MATERIALS AND METHODS

### Cell culture and reagents

The human osteosarcoma (U2OS) cells were grown in Dulbecco's Modified Eagle Medium (DMEM) supplemented with 10% fetal bovine serum, 2 mM L-glutamine, non-essential amino acids and penicillin/streptomycin at 37°C in a humidified incubator containing 5% CO_2_. Cells and cell culture reagents were obtained from the Tissue Culture facility of Fox Chase Cancer Center. Etoposide, ICRF193 and other chemicals were purchased from Sigma-Aldrich (MO, USA) unless otherwise indicated. Rabbit antibodies against *Xenopus* DNA2 and human DNA2 were prepared as described before (11). Other antibodies used in the study are: mouse anti-RPA1 monoclonal (Calbiochem-EMD Millipore, CA, USA), mouse anti-RPA2 monoclonal (Calbiochem-EMD Millipore, CA, USA), rabbit anti-TOP2α polyclonal (Bethyl, TX, USA), rabbit anti-MRE11 polyclonal (Abcam, MA, USA), mouse anti-RAD50 monoclonal (GeneTex, CA, USA), mouse anti-CtIP monoclonal (Active Motif, CA, USA), rabbit anti-EXO1 polyclonal (Protein Tech, IL, USA), rabbit anti-CenpF polyclonal (Dr Timothy Yen) and rabbit anti-BLM polyclonal (Dr Norma Neff). Click-iT EdU Alexa Fluor 647 imaging kit with Hoechst, goat anti-mouse Alexa Fluor 488 and goat anti-rabbit Alexa Fluor 568 were purchased from Invitrogen (CA, USA). DNA2 siRNAs (M-026431–00 and D-026431–03) and control siRNA (D-0012101–03) were purchased from GE Dharmacon-Fisher Scientific (PA, USA).

### Establishment of stable cells expressing DNA2

The DNA2 open reading frame (ORF) was subcloned downstream of the CMV promoter in a vector (pDs-NA) that carries the kanamycin resistance gene. The plasmid was digested with the restriction enzyme BsaI, which has a unique site outside the kanamycin resistance gene and the DNA2 gene, and then transfected into U2OS cells. Stable cells expressing DNA2 were selected with antibiotic G418 at 1 mg/ml concentration. The siRNA resistant version of DNA2 was constructed by introducing silent mutations in the siRNA target sequence. Stable cells expressing this gene were constructed in the same way as for the original DNA2 gene.

### Knockdown of DNA2 by siRNAs

U2OS cells were seeded in 24-well plates containing coverslips at a density of 4000 cells per well. After 24 h of incubation, cells were transfected with 20 nM of siRNAs using HiPerFect (Qiagen, CA, USA) following the manufacturer's protocol. This step was repeated 24 h later and cells were used for experiments after another 48 h. The efficiency of siRNA knockdown was determined by western blot. For DNA2, the endogenous level was below detection by western blot, so the efficiency was evaluated in stable cells that ectopically expressed DNA2. The effect of DNA2 siRNAs on other proteins was evaluated in normal U2OS cells. For the partial knockdown of DNA2, the concentration of siRNA was reduced to 10 nM and only one round of siRNA was applied for 48 h.

### Indirect immunofluorescence staining

U2OS cells were seeded at 4000 cells per well in 24-well plates containing coverslips. After siRNA treatment, 250 μM etoposide was added to the media. In experiments that followed DNA synthesis, EdU was added 15 min prior to etoposide. After 2 h of etoposide treatment, cells were pre-extracted with 0.1M PIPES (pH6.9)/1 mM EGTA/4M glycerol/0.2% Triton X-100 for 1 min, washed with 0.1M PIPES(pH6.9)/1 mM EGTA/4M glycerol for 2 min and fixed with 3.7% formaldehyde/50 mM PIPES (pH6.9)/1 mM MgCl_2_/5 mM EGTA for 20 min. For the nuclease sensitivity experiments, the extracted cells were incubated with RecJ or *Escherichia coli* Exo1 (NEB, MA, USA) at 37°C for 2 h before fixation. The fixed cells were then stained with the appropriate antibodies or EdU as previously described (27). Images were collected with a monochrome DAGE-MTI cooled CCD-300-RT camera (Scion Corp, MD, USA) and processed for proper contrast/level and pseudo-colors in Photoshop CS 4.0 (Adobe Systems, CA, USA).

### Colony formation assays

U2OS cells were seeded in a 24-well plate at 8000 cells/well. 24 h later, cells were treated with 10 nM control siRNA or DNA2 siRNA for 30 h. They were then reseeded into 6 well plates at 1000 cells/well and incubated for another 18 h. Etoposide was added to each well at final concentrations of 250, 50, 10, 2, 0.4 and 0 μM. After 2 h of treatment, etoposide was removed by washing five times with 2 ml of warm media per well. The plates were incubated for 9 more days and then stained with crystal violet to visualize colonies. Colonies were counted and the averages and standard deviations of the percentages of colonies of the no drug control were calculated and plotted. For comparisons of averages, a one-tailed T-test was conducted at 95% confidence level (c.l.).

### Preparation of *Xenopus* egg extracts and immunodepletion of *Xenopus* DNA2

Membrane-free cytosol derived from unfertilized interphase *Xenopus* eggs was prepared following the published protocol (37). To deplete DNA2, cytosol (40 μl + 20 μl ELB (10 mM HEPES (pH7.5), 250 mM sucrose, 2.5 mM MgCl_2_, 50 mM KCl, 1mM DTT)) was incubated with 20 μl Protein A Sepharose beads pre-coated with 80 μl of the rabbit anti-DNA2 serum or no serum at 4ºC for 1.5hr. The depletion was repeated and the depleted cytosol was saved as 5 μl aliquots at –80ºC.

### DNA resection assays in *Xenopus* egg extracts

The DNA substrates were prepared by amplifying a 5.7kb plasmid using Pfu DNA polymerase (Promega, WI, USA) and oligonucleotides carrying 5′-phosphotyrosine, biotin or hydroxyl groups (Midland, TX, USA) in the presence of ^32^P-labeled dATP. The products were purified first by Qiagen's polymerase chain reaction (PCR) purification columns and then by gel-filtration with Sepharose CL-2B beads (Sigma-Aldrich, MO, USA). The peak fractions were pooled and concentrated to 50 ng/μl. The 5′ avidin DNA was prepared by pre-incubating at 20 ng/μl of 5′ biotin DNA with 4 μg/μl Neutravidin (Pierce/ThermoScientific, IL, USA) for 10 min. A typical resection assay contained 5 μl depleted cytosol, 0.5 μl 10x ATP mix (20 mM ATP/200 mM phosphocreatine/0.5 mg/ml creatine kinase/50 mM DTT), 1–1.5 ng/μl DNA, and ELB buffer or DNA2 protein (total volume = 7.5 μl). The reactions were incubated at 22ºC and samples were taken at the indicated times and mixed with an equal volume of 2% SDS/25 mM EDTA. At the end, samples were brought up to 10 μl with H_2_O and treated with 1 μl proteinase K (10 mg/ml) at 22ºC for 2 h. The resection products were separated by 1% TAE/agarose gel electrophoresis and the gels were dried and exposed to Phosphorimager (Fuji) and film.

### Analysis of resection intermediates

DNA intermediates were isolated from *Xenopus* egg extracts by first incubating with 3 volumes of ELB buffer supplemented with 25 mM EDTA and 1/2 volume of proteinase K (10 mg/ml in H_2_O) at 22°C for 2 h and then purified with the PCR purification columns according to the manufacturer's protocol (Qiagen, CA, USA). To detect the presence of 5′ biotin, DNA was first incubated with ELB buffer or avidin on ice for 5 min, and then treated with T7 Exo (0.6 unit/μl; NEB, MA, USA) at 22ºC for 60 min. The products were analyzed by 1% TAE-agarose gel electrophoresis and the gels were first stained with SYBR Gold (Invitrogen, CA, USA) and then dried for exposure to film.

## RESULTS

### Etoposide-induced DSBs are efficiently resected into 3′ ss-DNA

**Figure 1.**
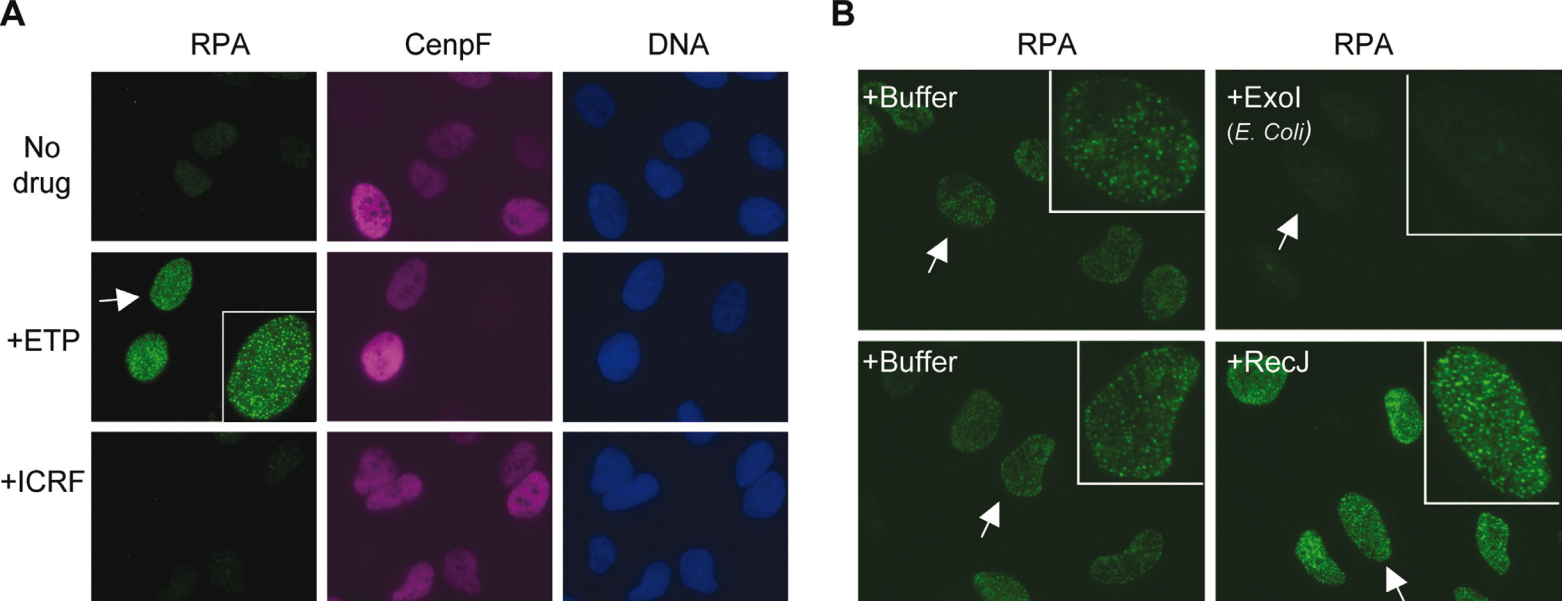
Etoposide-induced RPA foci represent 3′ ss-DNA resected from DSBs. (**A**) Etoposide but not ICRF193 induces RPA foci in U2OS cells. U2OS cells were treated with 250 μM etoposide or ICRF193 for 2 h, fixed, and stained for RPA and CenpF. (**B**) Etoposide-induced RPA foci are sensitive to 3′->5′ ss-DNA exonuclease. U2OS cells were treated with 250 μM etoposide for 2 h, permeabilized, incubated with RecJ (5′->3′ ss-exonuclease) or *E.coli* Exo1 (3′->5′ ss-exonuclease) for 2 h, and finally fixed and stained for RPA.

Etoposide induces a large number of discrete RPA foci in a TOP2-dependent way in the nucleus of S and G2 cells (27), but the exact nature of these foci is yet to be determined. It is known that etoposide-trapped TOP2ccs are converted into DSBs and DSBs can be resected into single-strand DNA during S and G2 phases, thus these RPA foci most likely represent RPA molecules bound to the ss-DNA of resected DSBs. To test this hypothesis, we first determined if RPA focus formation requires DSB induction or the mere inhibition of TOP2 activity. We compared etoposide with ICRF193, a different inhibitor of TOP2 that traps the enzyme on DNA after the resealing of the nicks (38). U2OS cells were treated with either etoposide or ICRF193 for 2 h and then fixed and stained for RPA and CenpF, a centromeric protein that accumulates in S phase and G2 cells (27,39). As shown in Figure 1A, while etoposide, as expected, induced a large number of RPA foci in S and G2 cells (CenpF^+^), ICRF193 did not. This suggests that the formation of RPA foci depends on the induction of DSBs rather than the mere inhibition of TOP2 activity.

If these RPA foci represent the ss-DNA generated by the resection of DSBs, one would predict that the ss-DNA is 3′ instead of 5′ based on the directionality of resection in yeast and *Xenopus* egg extracts. We tested this prediction by determining the sensitivity of RPA foci to two strand-specific ss-DNA exonucleases: *E. coli* Exo1, which degrades 3′ ss-DNA, and RecJ, which degrades 5′ ss-DNA. Cells were first treated with etoposide for 2 h, permeabilized with a Triton-X100 detergent-containing buffer, and then incubated with the two nucleases or their respective buffers. After 2 h of nuclease treatment, the nuclei were fixed for staining with anti-RPA2 antibodies. As shown in Figure 1B, *E. coli* Exo1 completely removed RPA foci, but RecJ did not have a significant effect. Taken together, these data strongly suggest that etoposide-induced RPA foci indeed represent the 3′ ss-DNA generated by the resection of DSBs. Detecting RPA foci can thus provide a convenient way for studying the resection of etoposide-induced DSBs in human cells.

### DNA2 knockdown by siRNAs inhibits the resection of etoposide-induced DSBs

**Figure 2.**
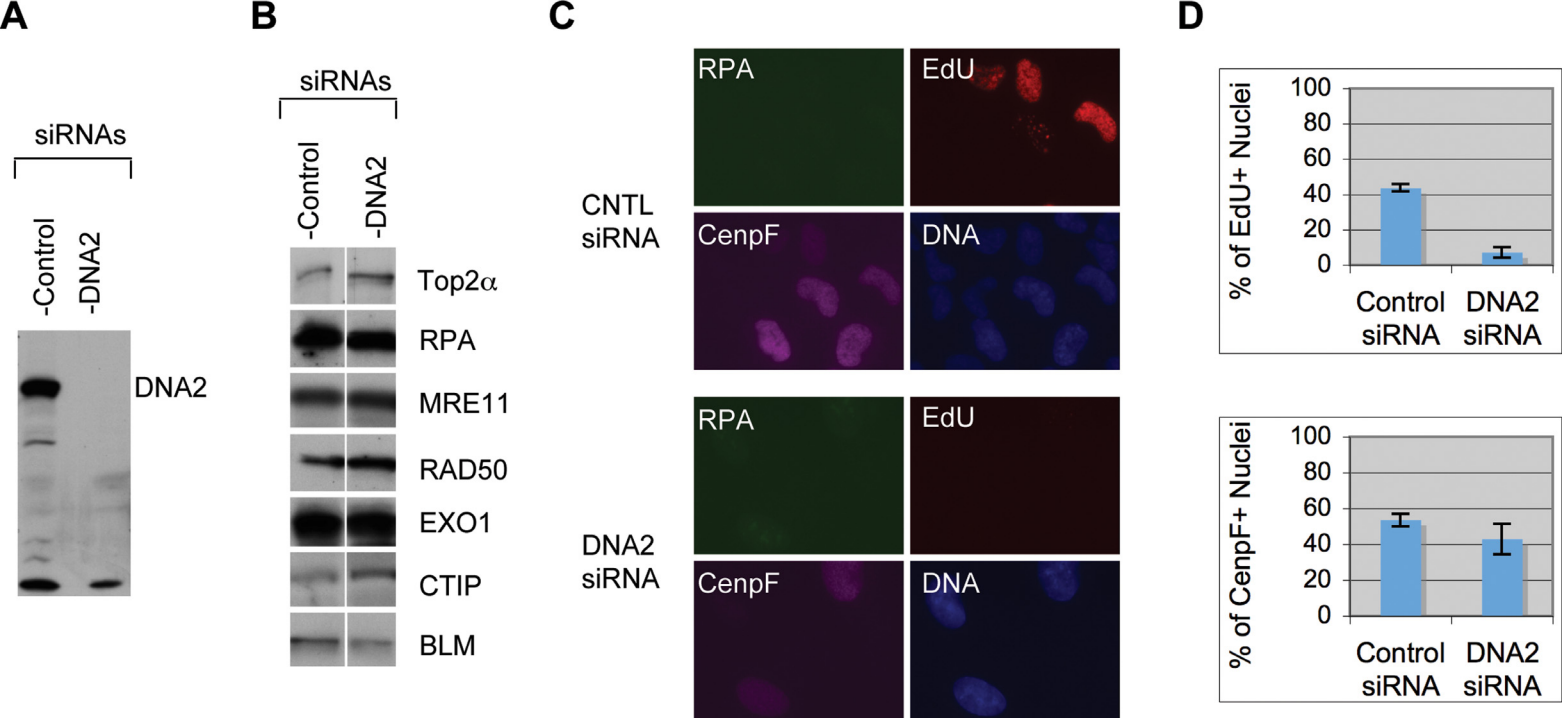
Effect of DNA2 siRNA on cell proliferation. U2OS cells were treated with two rounds of 20 nM control or DNA2 siRNA for 72 h and then subjected to various analyses. (**A**) Western blot of DNA2. (**B**) Western blot of other resection proteins. (**C**) Immunofluorescence staining of the siRNA-treated cells exposed to ETP. (**D**) Plots of the percentages of EdU+ cells and CenpF+ cells. Over 100 nuclei were counted and the percentages from three experiments were used for calculations of the averages and standard deviations.

**Figure 3.**
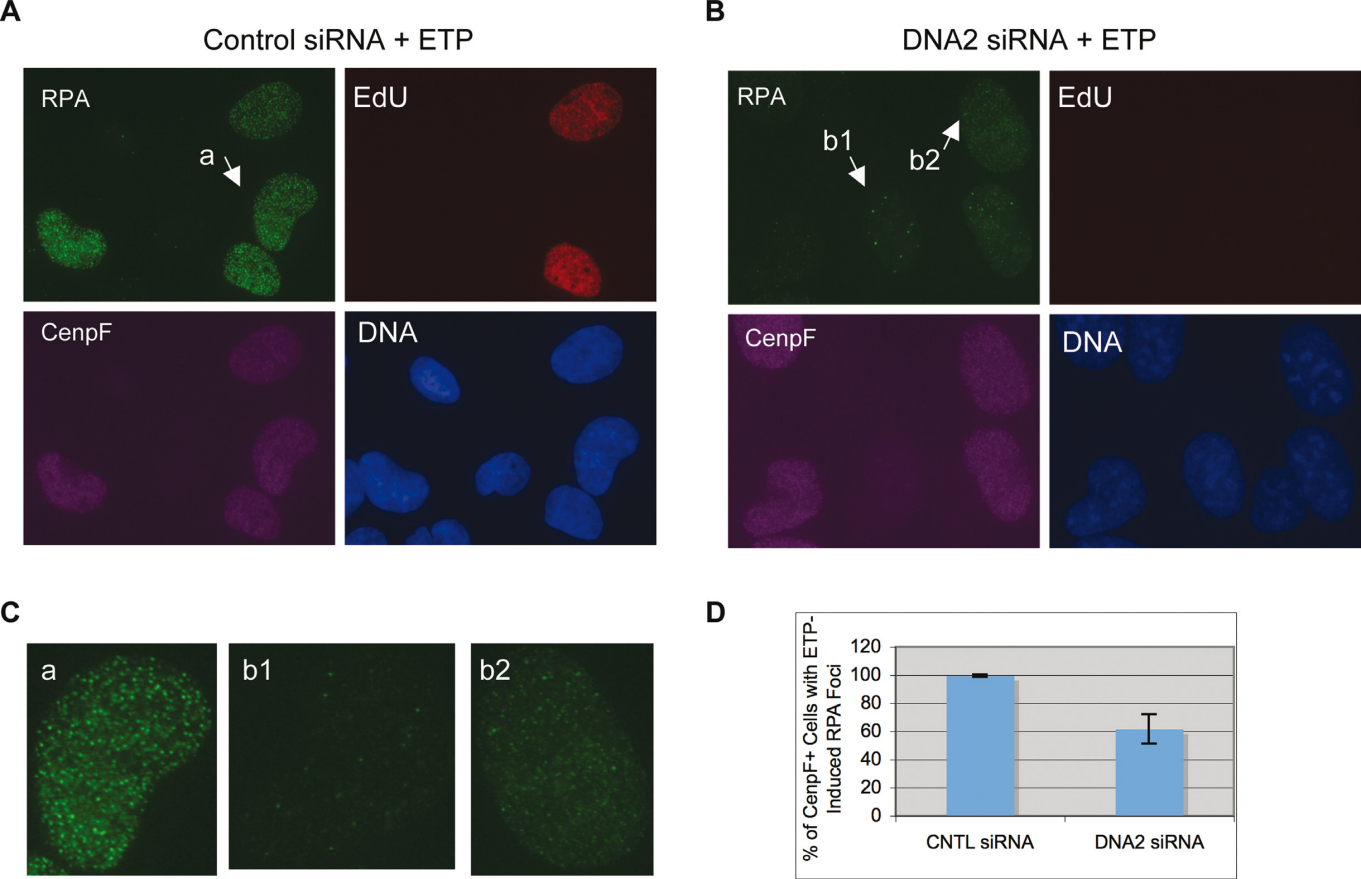
DNA2 siRNA causes a partial inhibition of RPA focus induction by etoposide. Cells were treated with two rounds of 20 nM control siRNA (**A**) or DNA2 siRNA (**B**) for 72 h, exposed to 250 μM etoposide for 2 h and finally fixed and stained for RPA, EdU and CenpF. (**C**) Close-ups of the nuclei indicated in (A) and (B). (**D**) The percentages of CenpF+ cells with etoposide-induced RPA foci were quantified and plotted. Over 100 nuclei were counted and the percentages from nine experiments were used for calculations of the averages and standard deviations.

What might be the major nuclease for the resection of etoposide induced DSBs? Studies in yeast and *Xenopus* egg extracts have shown that the major nuclease for 5′ strand resection of DSBs is the DNA2 protein (11,13). However, the DSBs in those studies are simple in structure, carrying either normal nucleotides or dideoxynucleotides. The etoposide-induced DSBs are expected to carry 5′ adducts, which might affect the choice of nuclease for resection. To determine if human DNA2 might be involved in the resection of etoposide-induced DSBs, we used siRNAs to specifically knock down its expression. As shown in Figure 2A and B, DNA2 could be efficiently knocked down without affecting the other resection proteins or TOP2α. The fraction of replicating cells was greatly reduced based on the staining for EdU, a nucleoside analog incorporated into DNA during replication (Figure 2C). However, about half of the cells were positive for CenpF staining, suggesting that they were arrested in S or G2 phase (Figure 2C and D). Because resection occurs in S and G2 cells, this means that DNA2's effect on resection can still be analyzed. The siRNA-treated cells were incubated with 250 μM etoposide for 2 h, fixed and stained for RPA, CenpF and EdU. In control siRNA-treated cells, as expected, all CenpF^+^ cells formed a large number of RPA foci, indicating efficient resection of DSBs (Figure 3A, C and D). For DNA2 siRNA-treated cells, however, 41% of the CenpF^+^ cells lacked or had greatly reduced etoposide-induced RPA foci (Figure 3B, C and D). The remaining nuclei showed etoposide-induced RPA foci, but usually fainter in intensity than those in control siRNA-treated cells. Etoposide can induce DSBs with 5′ adducts as well as DSBs with clean ends, so the resection defect is general rather than unique to a particular type of DSB.

**Figure 4.**
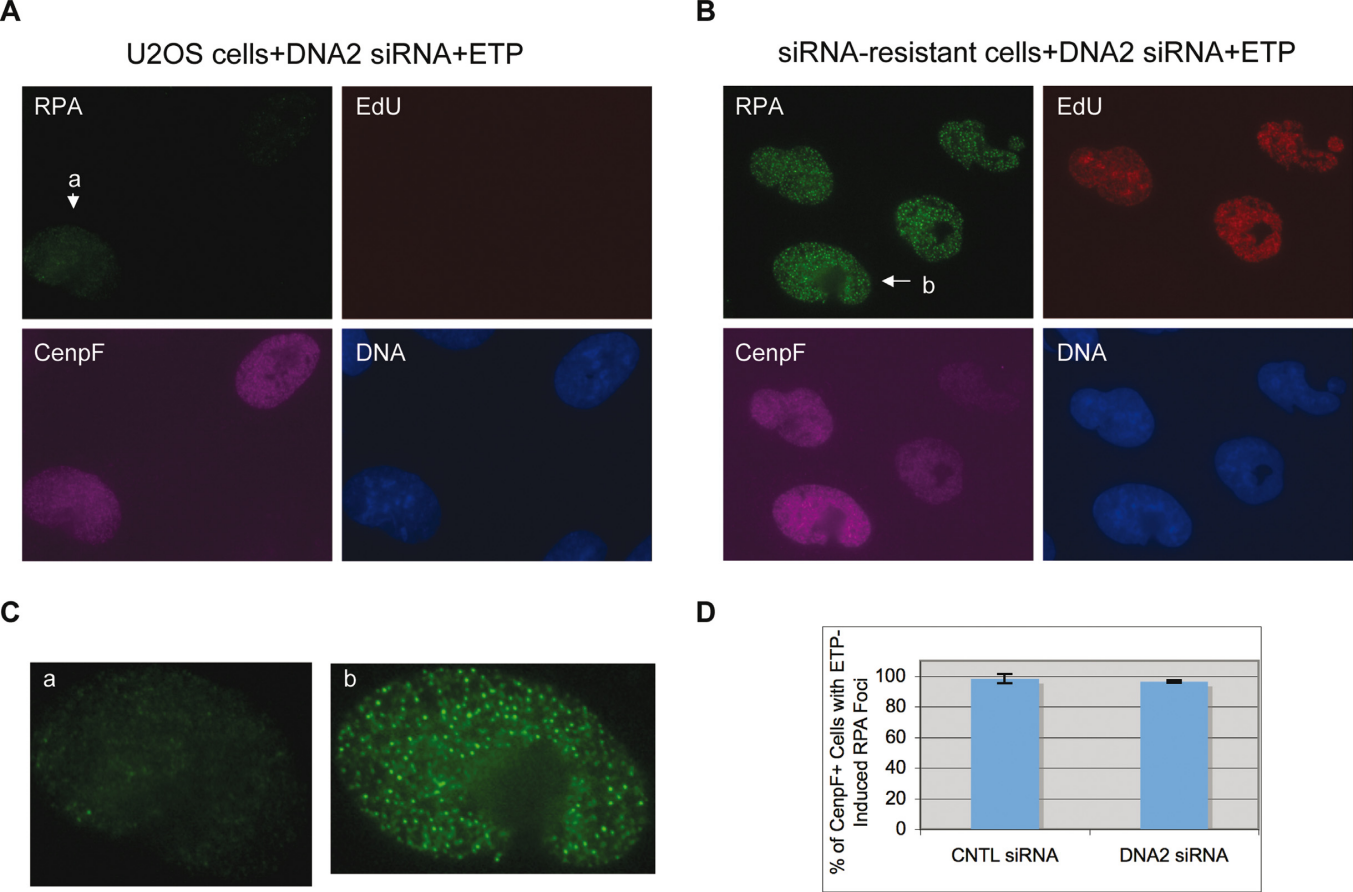
Complementation of DNA2 siRNA's effect by the siRNA-resistant DNA2 gene. U2OS cells (**A**) and U2OS cells expressing the siRNA-resistant DNA2 gene (**B**) were treated with two rounds of 20 nM DNA2 siRNA for a total of 72 h, then exposed to 250 μM etoposide and finally fixed and stained for RPA, EdU and CenpF. (**C**) Close-ups of the nuclei indicated in (A) and (B). (**D**) The percentages of CenpF+ cells with etoposide-induced RPA foci were quantified and plotted. Over 100 nuclei were counted and the percentages from three experiments were used for calculations of the averages and standard deviations.

To confirm that this effect was specific rather than off-target, we constructed a cell line that expressed a siRNA-resistant version of DNA2 by introducing silent mutations into the siRNA target sequence. These cells were treated with the DNA2 siRNA and then etoposide. As shown in Figure 4, they formed normal etoposide-induced RPA foci. Collectively, these data suggest that DNA2 is a major nuclease for the resection of etoposide-induced DSBs in U2OS cells.

### Partial knockdown of DNA2 causes hypersensitivity to etoposide

**Figure 5.**
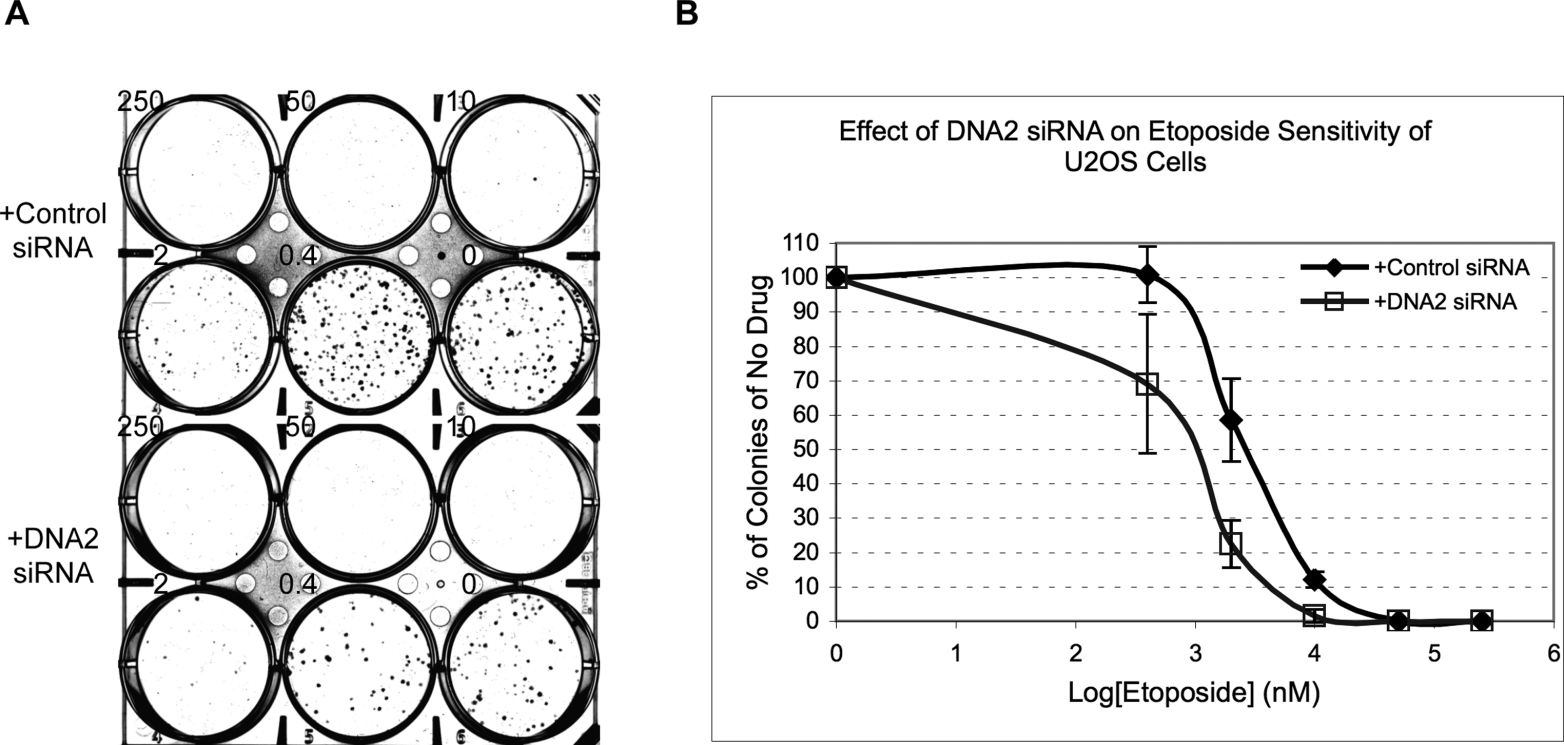
U2OS cells partially depleted of DNA2 are hyper-sensitive to etoposide. U2OS cells treated with 1 round of 10 nM control or DNA2 siRNA for 48 h were exposed to etoposide at the indicated concentrations for 2 h and then allowed to grow in fresh media for 9 more days. (**A**) The resulting colonies were stained with crystal violet. (**B**) The percentages of colonies relative to the no drug well were quantified and plotted. The averages and standard deviations were calculated from the data of three experiments.

DNA2's role in resection suggests that it might be important for the repair of etoposide-induced DSBs and consequently the sensitivity of cells to this drug. DNA2 is essential for cell proliferation, so to test this hypothesis we treated cells with a reduced amount of DNA2 siRNA and for only 48 h to partially knockdown DNA2. Cells were then treated with different concentrations of etoposide for 2 h, washed with media and incubated for 9 more days to allow surviving cells to form colonies. As shown in Figure 5, partial knockdown of DNA2 caused hyper-sensitivity to etoposide. At 2 μM of etoposide, only 22% of DNA2 siRNA-treated cells survived the drug to form colonies, which was significantly lower than the 59% of control siRNA-treated cells (*P*-value = 0.0085). These data suggest that DNA2 indeed plays an important role in DSB repair and cell survival after etoposide treatment.

### Depletion of DNA2 from *Xenopus* egg extracts inhibits resection of DSBs with 5′ adducts

**Figure 6.**
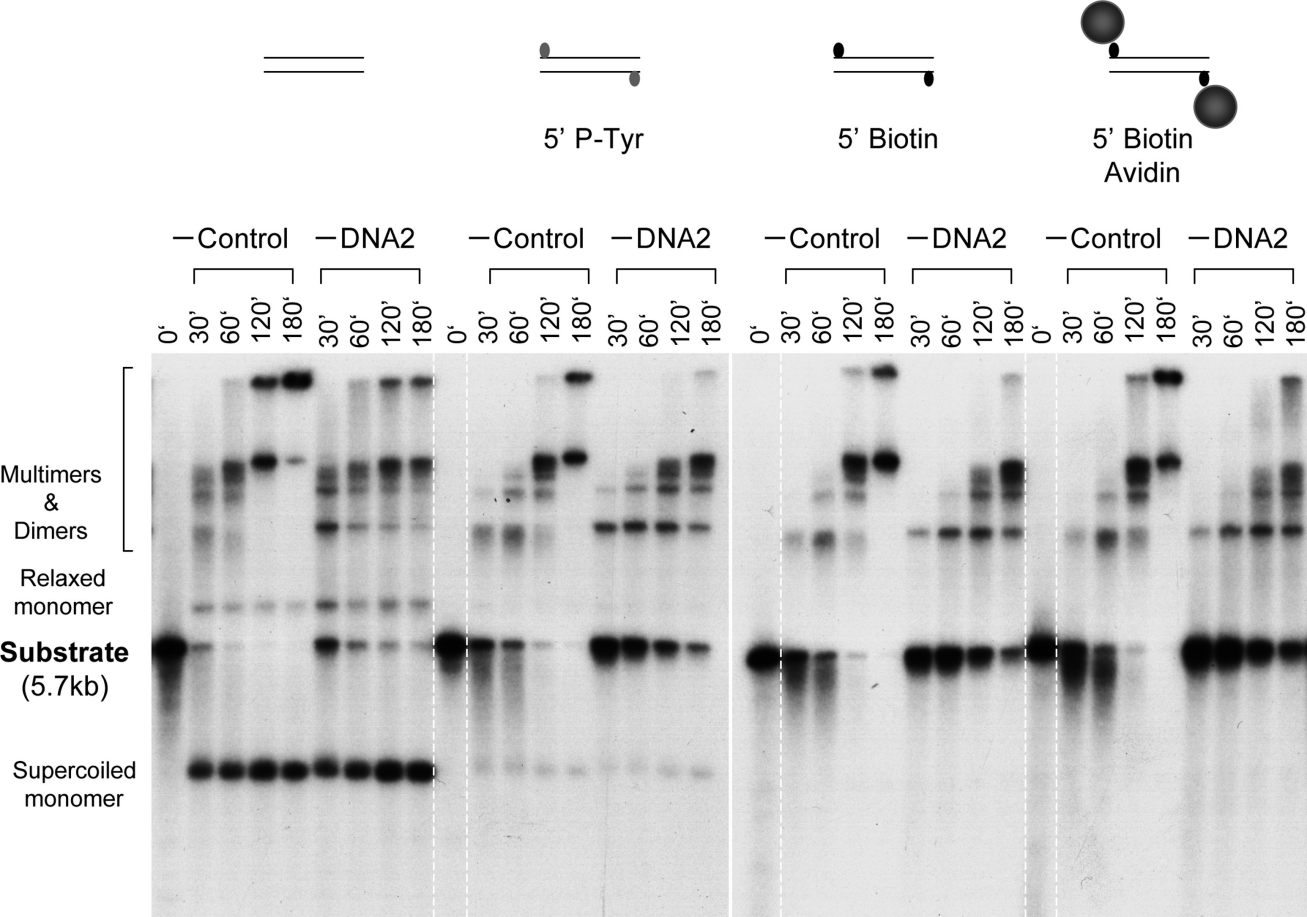
Depletion of DNA2 inhibits resection of DNA with 5′ adducts. (**A**) DNA substrates bearing different types of ends were incubated at 1.5 ng/μl with the control or DNA2-depleted extracts. Samples were treated with SDS-EDTA-Proteinase K, and separated on an 1% TAE-agarose gel. The gel was dried and exposed to X-ray film.

**Figure 7.**
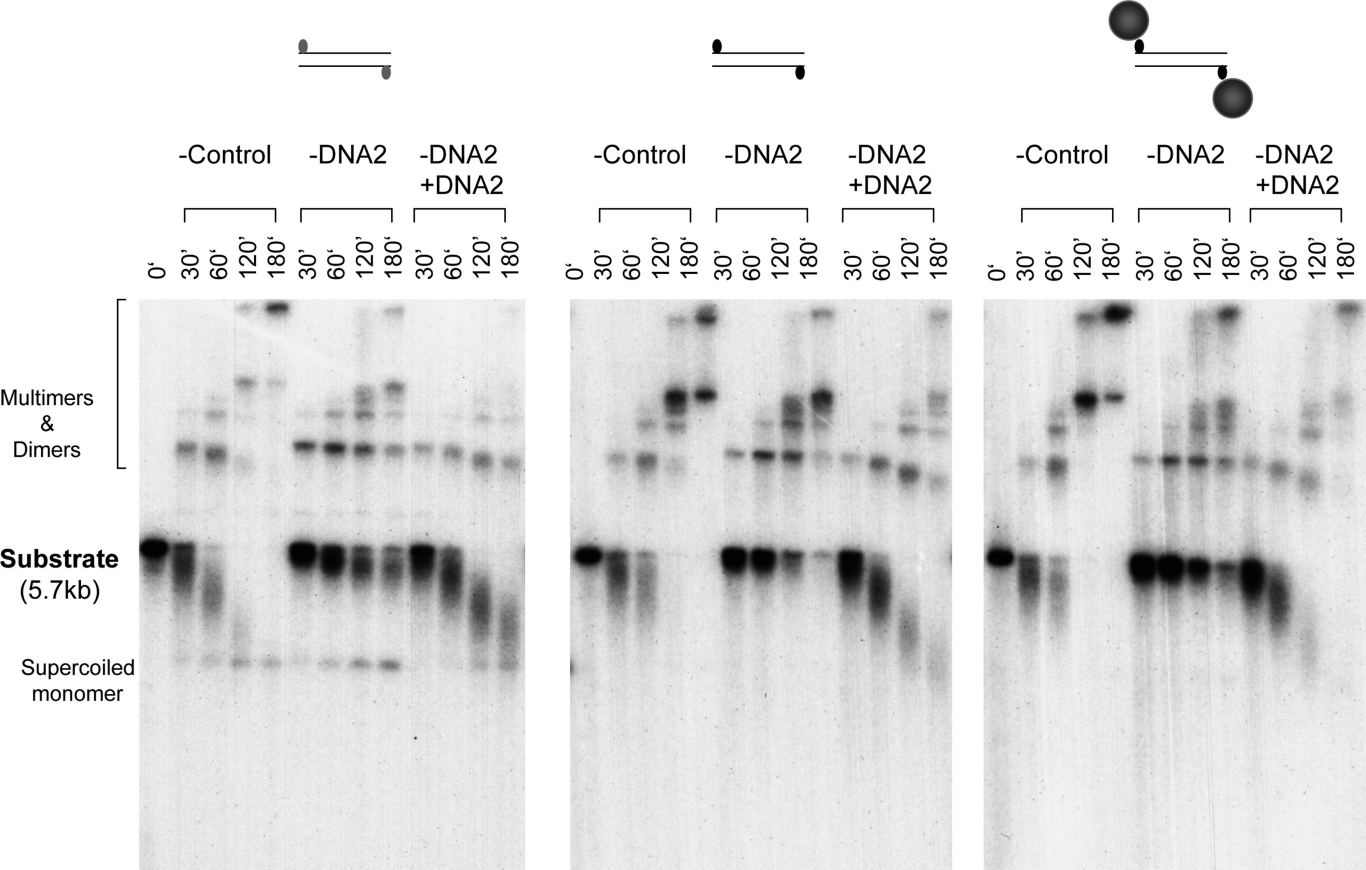
Rescue of the DNA2 depletion defect by the purified DNA2 protein. DNA substrates bearing different types of ends were incubated with the DNA2-depleted extracts supplemented with either buffer or DNA2 protein. Samples were treated with SDS-EDTA-Proteinase K, and separated on an 1% TAE-agarose gel. The gel was dried and exposed to X-ray film.

The cellular studies above suggest that DNA2 is a major enzyme for resecting etoposide-induced DSBs in cells. However, it does not necessarily mean that these are the DSBs that carry 5′ adducts. It is possible that etoposide also induces DSBs with clean ends (for example, via the cleavage of stalled replication forks) and these clean DSBs are the ones being resected. We have found that DNA substrates with different types of model adducts at the 5′ end are efficiently resected in *Xenopus* egg extracts (Liao et al., manuscript submitted). We thus used this system to more rigorously determine if the resection observed in *Xenopus* egg extracts is also dependent on DNA2. Depletion was carried out with Protein-A Sepharose beads coated with anti-DNA2 antibodies or control beads following the previous protocol (11). The depleted extracts were then incubated with various DNA substrates and samples taken at the indicated times were analyzed by gel electrophoresis. As shown in Figure 6, in mock-depleted extracts, DNA with 5′ -OH was efficiently repaired into supercoiled and relaxed monomers and some dimers and multimers. This is consistent with the model that such ends could simply be phosphorylated and religated by the NHEJ pathway. In contrast, for DNA with 5′ p-Tyr, which mimics degraded TOP2, there was a lot of degradation and much less repair products. DNA with 5′ biotin was very similar to DNA with 5′ p-Tyr except for that the amount of supercoiled monomer product was even less (below detection). For the DNA with 5′ avidin, which mimics intact TOP2, there was also extensive degradation and no detectable level of supercoiled monomer product. DNA2 depletion had no significant effect on the repair of 5′ -OH DNA, but caused a significant slowdown in the resection of the other three types of DNA. Even after 180 min of incubation, there were still some substrates left, especially for the 5′ avidin DNA. Resection of the 5′ avidin DNA was slightly slower than that of the 5′ p-Tyr or 5′ biotin DNA, most likely because avidin is a bulkier adduct, posing a more difficult physical obstacle for the loading of resection proteins. To confirm that this effect was specific rather than due to non-specific removal of other resection factors, we attempted to complement the DNA2-depleted extracts with the purified DNA2 protein. As shown in Figure 7, the purified DNA2 restored the resection activity to the DNA2-depleted extract for all three DNA substrates. Together these data demonstrate that DNA2 is indeed a major nuclease for the resection of DSBs with adducts at the 5′ end.

### DNA2 acts after the removal of the 5′ bulky adduct

**Figure 8.**
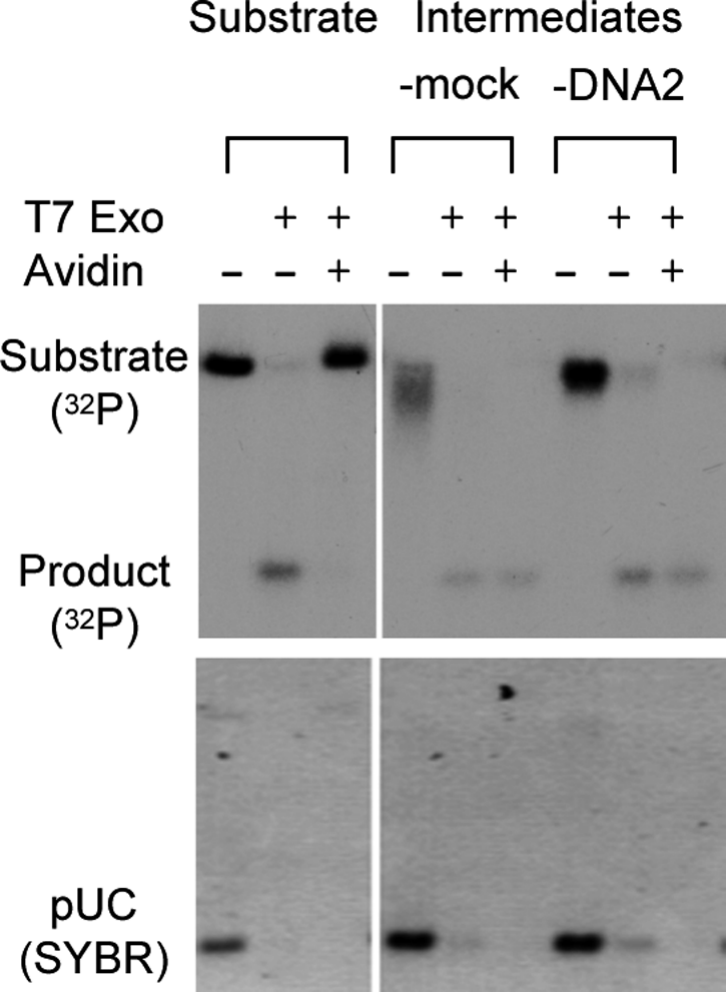
DNA2 does not affect the removal of avidin from the 5′ end. The resection intermediates were isolated after 30 min in the mock or DNA2-depleted extracts. They and the substrate were treated with T7 Exo in the presence of absence of avidin. The reactions also contained a linear plasmid (pUC) to serve as a control for digestion. The products were analyzed on a 1% TAE-agarose gel, stained with SYBR Gold, dried and exposed to X-ray film.

5′ bulky adducts pose a serious steric hindrance to the access of resection nucleases and helicases to the 5′ end. Enzymatic studies showed that in the presence of streptavidin, SAE2 activates the endonuclease activity of MRE11 to cleave internally the 5′ strand. However, it is unclear if these two proteins are sufficient in cells and whole extracts. An important mechanistic question is if DNA2, in addition to its role in long range resection, also plays a role in the initial removal of the 5′ adduct. To address this question, the DNA with 5′ avidin was re-isolated after incubation in the extracts and analyzed for the existence of the 5′ biotin using the bacterial T7 Exo nuclease, which degrades ds-DNA in the 5′->3′ direction. 5′ biotin per se did not inhibit the activity of T7 Exo (Figure 8). However, in the presence of avidin, the DNA became resistant to T7 Exo. Notably, the linear pBS DNA with no biotin at the end in the same reactions was equally sensitive to T7 Exo with or without avidin, indicating that it's the avidin at the 5′ end that is blocking T7 Exo. The DNA re-isolated after 30 min of incubation in the mock- or DNA2-depleted extracts were sensitive to T7 Exo (Figure 8). Pre-incubation with avidin did not protect them from T7 Exo, suggesting that they had lost the 5′ biotin and thus avidin. This suggests that DNA2 acts only after the removal of the 5′ bulky adduct.

## DISCUSSION

How DSBs with 5′ adducts such as those induced by the anti-cancer drug etoposide are repaired is an important question not only for basic mechanistic reasons but also for potential clinical applications. While TDP2-mediated NHEJ is a major pathway for repairing them, HDR is also implicated but poorly understood. In this study we have U2OS cells and *Xenopus* egg extracts to study the resection of etoposide-induced DSBs. Our major findings are: (i) etoposide-induced DSBs are efficiently resected into 3′ ss-DNA; (ii) DNA2 is a major nuclease for the resection of etoposide-induced DSBs; (iii) reduced levels of DNA2 cause cells to be hypersensitive to etoposide; (iv) DNA2 is also a major nuclease for resection of these DNA substrates in *Xenopus* egg extracts; and (v) DNA2 acts after the removal of the 5′ bulky adduct. Together these findings strongly suggest that DNA2-mediated resection is a major repair mechanism for DSBs with 5′ adducts.

Many DNA damaging agents induce discrete subnuclear RPA foci in cells. It is generally assumed that such RPA foci represent ss-DNA resulting from resection and are thus often used as markers for resection. However, other explanations are possible. For example, when replication forks stall, unwinding can continue and might also generate long-stretches of ss-DNA to which RPA molecules bind. In this case, the ss-DNA would be a bubble rather than have ends. Our experiment using strand-specific exonucleases demonstrated definitively that the RPA foci induced by etoposide represent 3′ ss-DNA, the expected product of DSB resection. To our knowledge, this is the first determination of the nature of RPA foci induced by DNA damaging agents in cells. In principle, the same technique can be applied to RPA foci induced by other agents, such as camptothecin, to determine if they are also the result of DSB resection.

Biochemical studies in *Xenopus* egg extracts and genetic analyses in yeast have shown that DNA2 is a major nuclease for the resection of DSBs. In human cells, observations in several studies are consistent with DNA2 also playing an important role in resection (40–42). However, these studies use camptothecin and/or cisplatin to induce DSBs, which require the collision of replication forks with trapped topoisomerase I (for campothecin) or interstrand crosslinks (for cisplatin). As shown by this and other studies, DNA2 is an essential gene and its knockdown by siRNAs inhibits DNA replication (41,43,44). It is thus difficult to conclude if the reported effect is direct on resection or indirect as the result of replication inhibition. Our study does not suffer from this ambiguity because it uses a high concentration of etoposide, which can induce DSBs in a replication-independent manner (27). It thus provides a more rigorous demonstration of DNA2's role in DSB resection in human cells. This conclusion is further validated by biochemical reconstitution experiments with model DNA substrates carrying defined 5′ adducts incubated in *Xenopus* egg extracts. *In vivo*, partial knockdown of DNA2 renders U2OS cells hypersensitive to etoposide, which, while not a direct proof, is consistent with a role for DNA2 in resection-mediated HDR repair. The observed effect is relatively modest but statistically significant. Considering that DNA2 is only partially knocked down, it is most likely an under-estimation of the contribution of DNA2 to the repair of etoposide-induced DSBs.

While our study shows that DNA2 plays an important role in the resection of etoposide-induced DSBs, it is unlikely to be the only protein involved. In fact, a majority of the cells still show RPA foci, albeit generally fainter than those in control cells. MRN, CtIP, EXO1 and RecQ type-helicases BLM and WRN have also been shown to be involved in DSB resection (16,17,45). Understanding the mechanistic relationship among these different proteins in the resection of etoposide-induced DSBs would be an important topic for future research. Of particular interest is the relative contribution of different resection proteins as well as TDP2 to the repair of etoposide-induced DSBs. It is known that the efficacy of etoposide varies among different types of cancer. Conceivably, this variability is at least partially the result of differential expression of TDP2, DNA2 and various resection proteins. Analyzing the expression of these proteins in different cancer cells might identify biomarkers for the optimal use of etoposide in cancer therapy.
